# Drought and Heat Differentially Affect XTH Expression and XET Activity and Action in 3-Day-Old Seedlings of Durum Wheat Cultivars with Different Stress Susceptibility

**DOI:** 10.3389/fpls.2016.01686

**Published:** 2016-11-10

**Authors:** Andrea Iurlaro, Monica De Caroli, Erika Sabella, Mariarosaria De Pascali, Patrizia Rampino, Luigi De Bellis, Carla Perrotta, Giuseppe Dalessandro, Gabriella Piro, Stephen C. Fry, Marcello S. Lenucci

**Affiliations:** ^1^Dipartimento di Scienze e Tecnologie Biologiche ed Ambientali, Università del SalentoLecce, Italy; ^2^The Edinburgh Cell Wall Group, Institute of Molecular Plant Sciences, The University of EdinburghEdinburgh, UK

**Keywords:** climate change, high temperature, plant cell wall, *Triticum durum*, water deficit, xyloglucan endotransglucosylase/hydrolase

## Abstract

Heat and drought stress have emerged as major constraints for durum wheat production. In the Mediterranean area, their negative effect on crop productivity is expected to be exacerbated by the occurring climate change. Xyloglucan endotransglucosylase/hydrolases (XTHs) are chief enzymes in cell wall remodeling, whose relevance in cell expansion and morphogenesis suggests a central role in stress responses. In this work the potential role of XTHs in abiotic stress tolerance was investigated in durum wheat. The separate effects of dehydration and heat exposure on XTH expression and its endotransglucosylase (XET) *in vitro* activity and *in vivo* action have been monitored, up to 24 h, in the apical and sub-apical root regions and shoots excised from 3-day-old seedlings of durum wheat cultivars differing in stress susceptibility/tolerance. Dehydration and heat stress differentially influence the XTH expression profiles and the activity and action of XET in the wheat seedlings, depending on the degree of susceptibility/tolerance of the cultivars, the organ, the topological region of the root and, within the root, on the gradient of cell differentiation. The root apical region was the zone mainly affected by both treatments in all assayed cultivars, while no change in XET activity was observed at shoot level, irrespective of susceptibility/tolerance, confirming the pivotal role of the root in stress perception, signaling, and response. Conflicting effects were observed depending on stress type: dehydration evoked an overall increase, at least in the apical region of the root, of XET activity and action, while a significant inhibition was caused by heat treatment in most cultivars. The data suggest that differential changes in XET action in defined portions of the root of young durum wheat seedlings may have a role as a response to drought and heat stress, thus contributing to seedling survival and crop establishment. A thorough understanding of the mechanisms underlying these variations could represent the theoretical basis for implementing breeding strategies to develop new highly productive hybrids adapted to future climate scenarios.

## Introduction

The extraordinary acceleration of human population growth in recent centuries is determining a fast-increasing demand for food, mainly cereals, and other agronomic crops. In recent decades, modern farming techniques and plant breeding programs enabled a linear enhancement in agricultural productivity; however, the positive trend is expected to slow down because global climate change is leading to adverse environmental conditions and to related negative effects on crop quality and yield (Lobell and Gourdji, 2012). The increase in global average temperature recorded in the 20th century was approximately 0.74°C; even worse perspectives are predicted by simulation models with temperature rising between 2.5 and 5.4°C by 2100, coupled with a decrease in precipitation of about 15%, if no mitigation efforts take place to reduce the emission of green-house gasses (Tadross et al., 2007; Ciscar, 2012). Dramatic changes in rainfall frequency, intensity, duration, and distribution have also been forecast at regional scale (Kao and Ganguly, 2011). This combination of events could significantly modify the levels of heat and drought stresses negatively impacting growth, development, and productivity of cultivated plants (Trenberth, 2011; Vadez et al., 2011).

Soil dryness and high temperature are main limitations to the productivity of agro-ecosystems. In cultivated plants, drought and heat stresses act at a metabolic level, negatively affecting agronomic performance. Seed germination and seedling growth rates are reduced, as well as yield, quality, and safety of the final product (Hasanuzzaman et al., 2013). In cereals, for example, a decrease of the kernel number inside the ear (Sekhon et al., 2010), of the number and size of starch granules and of the amount of lipids and proteins within the kernel (Wilhelm et al., 1999) has been reported under stress conditions, besides significant variations in the bromatological composition of flours (Wardlaw et al., 2002; Balla et al., 2011).

Drought and heat are multidimensional stresses that can hit the plant at various stages of the life cycle, within short (minutes) to prolonged (weeks or even months) time periods with consequences at molecular, physiological, and morphological level, leading to reduced plant growth, improper organ development, photosynthesis inhibition, severe cellular damage, and ultimately, death (Zhang et al., 2005; Wahid et al., 2007).

Plants exposed to sub-lethal abiotic stress conditions exhibit a broad range of morphogenic responses related, at least in part, to variations in gene expression and in the action of agents involved in organ development and growth processes. Despite the diversity of stress-related phenotypes, generic “stress-induced morphogenic responses” (SIMRs) have been recognized. SIMRs comprise alterations in cell division and elongation, processes probably functioning as focal points of response regulation (Potters et al., 2007, 2009).

The plant primary cell wall is a dynamic structure that plays a fundamental role in controlling cell growth and morphogenesis. Its three-dimensional architecture is dynamically remodeled by a number of enzymatic and non-enzymatic factors, in turn controlled by phytohormone-related physiological and stress-induced processes (Wolf et al., 2012). Among them, xyloglucan endotransglucosylase/hydrolase (XTH) enzymes catalyze either the cleavage and molecular grafting of the β-(1-4)-xyloglucan backbone through endotransglucosylase (XET) activity or its irreversible shortening through the endohydrolase (XEH) activity (Fry et al., 1992; Nishitani and Tominaga, 1992; Rose et al., 2002; Shi et al., 2015), thus having a role in the modification of the load-bearing cell wall framework (Nishitani and Vissenberg, 2007; Park and Cosgrove, 2015). The two (XET and XEH) known activities of XTH proteins have been monitored in growing tissues of monocots and dicots suggesting that these enzymes are ubiquitous and essential for the development of all land plants (Van Sandt et al., 2007a; Eklöf and Brumer, 2010; Franková and Fry, 2013). The extractable XET activity and XTH gene expression have often been correlated with cell expansion (Fry et al., 1992; Hetherington and Fry, 1993; Pritchard et al., 1993; Potter and Fry, 1994; Xu et al., 1995; Palmer and Davies, 1996; Antosiewicz et al., 1997; Catalá et al., 1997; Vissenberg et al., 2000, 2003, 2005; Van Sandt et al., 2007a), and clear evidence of the role of some XTHs as cell growth promoters has been established by loss- and gain-of-function molecular approaches (Osato et al., 2006; Van Sandt et al., 2007b; Zhu et al., 2012; Hara et al., 2014).

The relevance of the cell wall remodeling functions of XTH enzymes in the processes of cell division and/or expansion growth suggests a potential central role in stress responses. The *Arabidopsis* genome contains 33 different *XTH* genes showing diverse and distinct expression patterns in terms of organ specificity and response to developmental and environmental stimuli such as touch, darkness, cold/heat shock (Xu et al., 1995, 1996), wind (Antosiewicz et al., 1997), flooding (Saab and Sachs, 1995), drought and salt stresses (Schopfer and Liszkay, 2006), light quality (Sasidharan et al., 2010), or aluminum stress (Shi et al., 2015). A similar number of *XTH* genes was reported in other angiosperms including poplar (*Populus trichocarpa* Torr. & A.Gray ex. Hook., 41 members) and the monocots rice (*Oryza sativa* L., 29 members), and wheat (*Triticum aestivum* L., >57 members; Yokoyama et al., 2004; Geisler-Lee et al., 2006; Liu et al., 2007). The functional role of this enzyme family in drought stress conditions has been shown in tomato (*Solanum lycopersicum* L.) and *Arabidopsis* overexpressing *CaXTH3* gene from hot pepper (*Capsicum annuum* L.). The transgenic plants exhibited an increased tolerance to severe water deficit, confirming that XTH is involved in drought tolerance (Cho et al., 2006; Choi et al., 2011). Furthermore, transgenic *Arabidopsis* plants, overexpressing *AtXTH21*, showed an improved frost tolerance (Shi et al., 2014). Interestingly, an enhanced cadmium tolerance was evidenced in transgenic poplar (*P. alba*, L.) overexpressing *XTH* genes, likely to be related to a decreased uptake and storage capacity of the root. This was accompanied by an increased xyloglucan degradation activity, leading to a reduction of xyloglucan content in root cell walls (Han et al., 2014). XTH enzymes also seem to be involved in triggering stem and petiole elongation, primarily through cellular expansion, in response to a reduction in perceived blue or red light (Keuskamp et al., 2012; Sasidharan et al., 2014).

High temperature and drought stress have emerged as the major constraint for durum wheat (*Triticum durum* Desf.) production (Kumar et al., 2013). Although, in the Mediterranean area these two stresses usually occur simultaneously, this study reports the separate effects of dehydration and heat exposure on XTH expression as well as XET *in vitro* activity and *in vivo* action in apical and sub-apical root regions and shoots excised from 3-day-old seedlings of durum wheat (*T. durum* Desf.) cultivars (Svevo, Creso, Ardente, and Simeto) differing in stress susceptibility/tolerance.

There is an important distinction between enzyme ‘activity’ (assayed *in vitro*, under optimized conditions, usually after solubilisation of the enzyme) and enzyme ‘action’ (usually monitored *in vivo* in the walls of living plant cells; Fry, 2004). An XTH which (when extracted) exhibits high activity *in vitro* might exhibit low action *in vivo* – for example because the enzyme may not be physically co-localized with its potential substrate(s), the endogenous xyloglucan may be ‘end-capped’ with an unusual repeat-unit such as V [α-Xyl-(1 → 4)-α-Xyl-(1 → 6)-Glc; Franková and Fry, 2012], the apoplastic pH may be non-optimal *in vivo*, or the enzyme may be inactivated by immobilization on cellulose (Franková and Fry, 2013). Conversely, an enzyme may exhibit higher action *in vivo* than would have been predicted by its activity measured *in vitro* – for example because the apoplast may contain cofactors that were omitted from the *in vitro* assay (Takeda and Fry, 2004) or simply because a proportion of the wall enzyme was inextractable. Another important difference is that enzyme action assays conducted *in vivo* permit a better spatial resolution than is feasible with activity assays conducted on enzyme extracts *in vitro*. In the present work, we use a fluorescently labeled xyloglucan oligosaccharide (XXXGol-SR) as acceptor substrate. For activity assays *in vitro*, the transglycosylation reaction is monitored in an artificial reaction mixture containing solubilised XTH, donor substrate (xyloglucan) and acceptor substrate (XXXGol-SR). In contrast, for action assays *in vivo*, only XXXGol-SR is added, and the ability of endogenous XTHs to act on endogenous xyloglucan (as donor substrate) can thereby be monitored. In short, enzyme activity assays indicate potential enzyme action, which may or may not be implemented *in vivo*.

The ability of plants to tolerate abiotic stress varies at both inter- and intra-specific level; so that, understanding the mechanisms underlying this variability as well as differences/similarities in molecular responses to different stresses, could identify and implement innovative strategies to develop breeding programs aimed at obtaining new high-yield productive hybrids adapted to climate future scenarios (Wahid et al., 2007).

## Materials and Methods

### Assessment of Basal Thermo-Tolerance and Response to Dehydration

Certified caryopsides of 20 *T. durum* cultivars (Ardente, Cappelli, Ciccio, Cirillo, Claudio, Colosseo, Creso, Hmoul/Chabaa, Iride, Kofa, Lloyd, Meridiano, Messapia, Mohawk, Neodur, Ofanto, Parsifal, Rascon/Tarro, Simeto, Svevo) obtained from the Dipartimento delle Scienze del Suolo della Pianta e degli Alimenti (Di.S.S.P.A.) of Bari (Italy) and the Dipartimento di Scienze Agrarie of Bologna (Italy) were germinated in Perligran (Deutsche perlite, Dortmund, Germany). Ten-day-old seedlings, grown at 25°C, were used to evaluate basal thermotolerance, measuring cell membrane stability (CMS), and the response to water stress by measurement of leaf relative water content (RWC). In particular CMS test was performed as described in Rampino et al. (2009) on three replicates of each cultivar, according to the method of Fokar et al. (1998) for basal thermotolerance assessment. Leaf RWC was measured in well-watered 10-day-old seedlings (used as control) as well as in dehydrated seedlings; dehydration treatment was performed placing seedlings on dry filter paper for 24 h at room temperature (Rampino et al., 2006). RWC values were calculated according to the formula: RWC% = [(fresh weight–dry weight)/(turgid weight–dry weight)] × 100 (Barrs and Weatherley, 1962) and expressed as the mean value of 10 replicates for each cultivar.

### Plant Growth Conditions and Treatments

Durum wheat (*T. durum* Desf.) caryopsides of the cultivars Svevo, Creso, Simeto, and Ardente, chosen on the basis of their relative stress susceptibility/tolerance, were soaked for 2–3 h in running tap water, surface-sterilized with 0.6 % (v/v) commercial bleach solution for 10 min, then germinated and grown on Petri dishes (Ø = 15 cm) containing two layers of water-moistened Whatman No. 1 filter paper (Whatman Ltd., UK) at 22°C for 3 days in the dark, according to Leucci et al. (2008).

Uniform 3-day-old seedlings were carefully selected and subjected to dehydration or heat treatment. For dehydration, the seedlings were transferred on to Petri dishes (Ø = 15 cm) containing a well water-moistened (control) or dry (stressed) Whatman No. 1 filter paper. The seedlings were incubated in these conditions for 2, 4, and 24 h, at 22°C, in the dark. Heat stress was induced by transferring the 3-day-old seedlings on to Petri dishes (Ø = 15 cm) containing well water-moistened Whatman No. 1 filter paper and incubating at 42°C, in the dark, for 2, 4, and 24 h. The control seedlings were grown at 22°C, for 2, 4, and 24 h.

**FIGURE 1:**
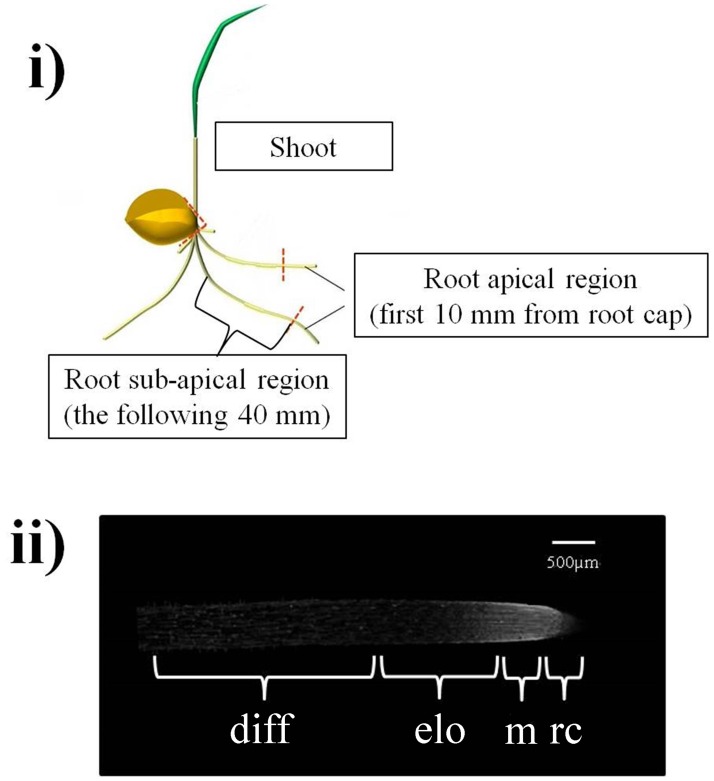
**(i)** Regions excised from wheat seedlings used for extraction of *XTH* transcripts and XET-active enzymes. At root level, the apical region (10 mm starting from the root cap) and the sub-apical region (the following 40 mm) were dissected and independently assayed. **(ii)** The region analyzed with confocal observations of *in situ* XET action corresponds to the first 5 mm of the root apical region and includes: the root cap (0–0.5 mm) – **rc**, the root meristem (between 0.5 and 1 mm) – **m**, the cell elongation zone (between 1 and 2.5 mm) – **elo** and the cell differentiation zone (between 2.5 and 5 mm) – **diff**.

At the end of the incubation period, three different segments [entire shoot (coleoptile and leaf), root sub-apical region, and root apical region (**Figure 1i**)] were excised from control and stressed seedling, immediately surface-dried on Whatman No. 1 filter paper, weighed and frozen in liquid nitrogen. The frozen samples were then freeze-dried (Lyophilizer Martin Christ, Osteride am Hanz, Germany) to constant weight (∼72 h).

### Identification of Durum Wheat XTH Coding Sequence

Two primers (XTH-for and XTH-rev) (Supplementary Table S1) were designed within highly conserved regions of known *XTH* sequences of related species (*T. aestivum* L., *Hordeum vulgare* L., *Bambusa oldhamii* Munro and *Zea mays* L.) and used to amplify the putative *XTH* fragments from a cDNA library of the roots of *T. durum*, cultivar Creso, obtained as described below (see section “Standard RNA Procedures and Quantitative Real-time PCR”). In this case the library was prepared starting from 2-cm-long apical root segments excised from 3-day-old seedlings not subjected to stress. The amplimer was cloned into pGEM-T Easy (Promega) and a single clone was checked by sequencing (Eurofins Genomics S.r.l., Milan, Italy) to confirm the specificity of the primers for XTHs in *T. durum*. The ExPASy Translate tool^1^ was used to deduce the amino acid sequence. Identity analysis was performed using the BLAST tool in GenBank.

### Standard RNA Procedures and Quantitative Real-Time PCR

Total RNA was isolated from the apical (10 mm starting from the root cap) and sub-apical (the next 40 mm) segment of the root excised from 3-day-old wheat seedlings subjected, and not, to different stress(es) for 0 (control), 2, and 4 h using SV Total RNA Isolation System according to the manufacturer’s instructions (PROMEGA). The RNA of each sample was reverse transcribed using oligo (dT) primer with TaqMan Reverse Transcription Reagents (Applied Biosystems^2^) according to the manufacturer’s standard protocol. The cDNAs obtained were quantified using a NanoDrop RND-1000 UV-visible spectrophotometer, diluted, and used for Real-Time PCR amplifications with specific primers (Supplementary Table S1). Quantitative real-time PCR was performed using SYBR Green fluorescent detection in a Real-Time PCR thermal cycler (ABI PRISM 7700 Sequence Detection System, Applied Biosystems) as previously reported (De Caroli et al., 2015). The PCR program was as follows: 3 min at 94°C; 35 cycles of 30 s at 94°C, 30 s at 60°C, and 30 s at 72°C; and 6 min at 75°C. The specificity of PCR products was checked in a melting-curve test with a heating rate of 0.2°C/s starting at 60°C. The melting profiles of the XTH quantitative real-time PCR products of control (**Supplementary Figure S1A**) and heat-stressed samples (**Supplementary Figure S1C**) showed a sharp single peak at a melting temperature (Tm) of 87.5°C for all cultivars, while those from drought-stressed samples (**Supplementary Figure S1B**) showed an additional peak at 83.5°C, regardless of cultivar, symptomatic of the formation of at least one other PCR product. The sharp and fully overlapping melting peaks provide an indication of the specificity of our primers for XTHs and also suggest the presence of a low level of polymorphism for XTHs expressed in the apical 50 mm segments of 3-day-old control and stressed wheat roots.

The α-*Tubulin* gene of *T. aestivum* (GeneBank accession number: U76558) was used as reference gene, as its variation coefficient, under stress conditions and during 0–4 h root growth, was found to be below 0.1 in *T. durum*, according to Czechowski et al. (2005), who described negligible variations under stress conditions in *Arabidopsis*. Tub-for and Tub-rev primers were used to amplify the selected reference gene (Supplementary Table S1).

Differences in gene expression between treated samples and the corresponding controls were considered biologically significant when the expression was at least doubled (≥twofold, up-regulation) or halved (≤-2, down-regulation) according to Chen et al. (2007).

### Enzyme Extraction

The freeze-dried samples were processed for enzyme extraction as reported in Fry et al. (2008). Briefly, samples were vigorously ground by pestle and mortar in a suitable amount (1:20 g freeze-dried matter/ml) of ice-cold extraction buffer [10 mM CaCl_2_, 300 mM succinate (Na^+^, pH 5.5), 20 mM ascorbate, 15% (v/v) glycerol, and 3% (w/v) polyvinylpolypyrrolidone]. The extraction was carried out on ice (4°C) for 2 h with occasional mixing. The extracts were filtered on Miracloth^®^ (EMD, Millipore, Billerica, MA, USA) and centrifuged at 12000 × *g* for 5 min. The supernatant (crude enzyme extract) was stored in single-use aliquots at -80°C. Total protein concentration was determined according to the Bradford (1976) method.

### Preparation of XXXGol-Sulforhodamine Conjugate (XXXGol-SR)

The xyloglucan heptasaccharide XXXG (10.62 mg; 10 μmol)) from Megazyme^3^ was reductively aminated to convert the reducing terminal glucose moiety to 1-amino-1-deoxyglucitol, and then fluorescently labeled by reaction of the amino group with lissamine rhodamine sulfonyl chloride to form a stable sulphonamide conjugate (XXXGol-SR) according to Fry (1997).

### Biochemical Assay of Extracted XET Activity

The XTH activity assay was modified from the method of Fry (1997). The crude enzyme extracts were used to test the XET activity in the different portions of the control and stressed seedlings. A reaction mixture formed by mixing crude XTH extract (10 μl), 1% tamarind xyloglucan solution (5 μl) and 6 μM XXXGol-SR (5 μl) was incubated for 18 h in the dark at room temperature (22°C). To eliminate potential artifacts during the assay, each crude enzyme extract (10 μl) was mixed with 5 μl of distilled water (instead of the xyloglucan solution) and with 5 μl of XXXGol-SR. After 18 h incubation, 5 μl of the reaction mixture was dried on a Whatman filter paper No. 3 (Whatman Ltd., UK). To de-stain the background, the paper was washed under running water for 16 h. The dried paper was examined under an ultraviolet lamp (λ = 254 nm). XET activity of the extracted XTH is indicated by a pale pink spot which emits an intense orange fluorescence. Images were acquired with a digital camera (Coolpix 8800, Nikon) in a dark room at the highest resolution (8.0 Megapixel). Quantification of fluorescence emitted by each sample was done using the shareware software of digital image processing ImageJ (Wayne Rasband, National Institutes of Health, USA).

### *In vivo* Assay of XET Action

The *in vivo* XET action of endogenous XTHs on endogenous xyloglucan (donor substrate) was evaluated in the roots of 3-day-old seedlings as described by Vissenberg et al. (2003). For dehydration treatment, the whole seedlings were dehydrated on filter paper, as described in Section “Plant Growth Conditions and Treatments,” for 2, 4, and 24 h and then incubated with the roots submerged in 3 mL of XXXGol-SR (6.5 μM) in water for 2 h on an orbital shaker (Edmund Bühler ks-15 Control, Hechingen, Germany), at low speed (50 rpm) to avoid mechanical damage of the roots but ensure a uniform labeling of the roots. The same procedure was used for well-watered control seedlings.

For heat stress, whole seedlings were incubated with the roots submerged in 6.5 μM XXXGol-SR for 2, 4, and 24 h at 42°C on an orbital shaker (50 rpm). Control seedlings were incubated as above but at 22°C. The incubation was followed by a 10-min wash in ethanol/formic acid/water (15:1:4, by vol.) and an incubation overnight in 5% (w/v) formic acid. Control and treated 5-mm apical root segments were excised with a razor blade (**Figure 1ii**), placed on a glass slide and immediately examined with a LSM 710 confocal laser scanning microscope (Zeiss, Oberkochen, Germany). To detect the sulforhodamine fluorescence, a 488-nm argon ion laser line was used, and the emission was recorded with a 581–589 nm filter set. All observations were performed with an N-Achroplan 10x/0.25 M27 objective (Zeiss). Multiple z-stack confocal sections were acquired on a identical thickness of the root (from the superficial to the equatorial portion). Projections of multiple z-stack confocal sections into one image were performed by generating a maximum intensity projection (Zen 2012 software, Zeiss). The power of laser line, the master gain, the digital gain, and the digital offset were identical for each experiment so that the images were comparable as reported in De Caroli et al. (2014). Quantification of fluorescence was done using the ImageJ software.

### Statistical Analysis

Results are presented as the mean value ± standard deviation of *n* independent replicate experiments. Statistical analysis was based on a one-way ANOVA test. Tukey’s *post hoc* method was applied to establish significant differences between means (*p* < 0.05). All statistical comparisons were performed using SigmaStat version 11.0 software (Systat Software Inc., Chicago, IL, USA).

## Results

### Evaluation of Relative Susceptibility/Tolerance to Dehydration and Thermal Treatment of a Selection of Durum Wheat Cultivars

In order to establish a ranking in phenotype susceptibility/tolerance to dehydration and thermal treatment, 10-day-old seedlings from a collection of 20 tetraploid wheat cultivars (T. durum Desf.) were analyzed at the physiological level using the RWC and CMS tests (Rampino et al., 2006, 2009).

**FIGURE 2:**
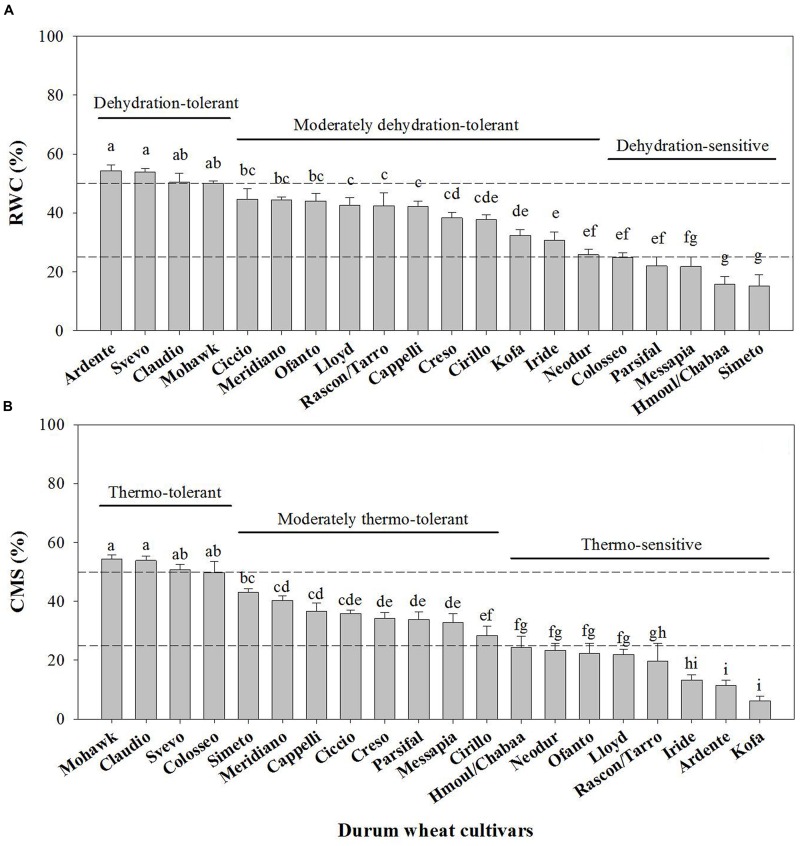
**Relative Water Content (RWC) (A)** and Cell Membrane Stability (CMS) **(B)** indices of *T. durum* Desf. cultivars. The reported RWC percentages refer to 10-day-old seedlings subjected to 24 h dehydration. RWCs of control plants (not shown) were not significantly different among the analyzed cultivars (RWC ∼98%). CMS indices were determined according to the method of Fokar et al. (1998) for basal thermo-tolerance assessment. Data are the mean ± standard deviation of three independent replicates (*n* = 3). Data were submitted to one-way analysis of variance (ANOVA), Different letters above bars indicate statistically significant differences among cultivars (*p* < 0.05), as determined by the Tukey’s *post hoc* test.

The results (**Figures 2A,B**) indicated a high variability in dehydration (*P* < 0.001; *F* = 69.917, *n* = 19) and heat treatment (*P* < 0.001; *F* = 83.139, *n* = 19) tolerance among the analyzed genotypes. In particular, we considered “sensitive” the cultivars exhibiting RWC or CMS values below 25%, moderately tolerant those with values between 50 and 25%, and “tolerant” those exceeding 50%, when subjected to dehydration or heat. According to this assumption and based on their relative stress susceptibility/tolerance, the Simeto (15.2 ± 3.7% RWC), Creso (38.3 ± 1.9% RWC), and Ardente (54.3 ± 1.9% RWC) cultivars, respectively, sensitive, moderately tolerant and tolerant to dehydration, and Ardente (11.4 ± 1.8% CMS), Creso (34.3 ± 2.0% CMS), and Svevo (50.8 ± 1.8% CMS) cultivars, respectively, sensitive, moderately tolerant and tolerant to thermal treatment, where selected and used in the subsequent phases of the research.

### Identification of Durum Wheat XTH Coding Sequence

Since the XTH coding sequences of durum wheat had not been reported, the primers XTH-for and XTH-rev (Supplementary Table S1), designed within highly conserved regions of known *XTH* sequences of related species (*T. aestivum* L., *H. vulgare* L., *B. oldhamii* Munro, and *Z. mays* L.), were used to amplify putative *XTH* fragments from a cDNA library of the roots of *T. durum* seedlings, cultivar Creso. An amplimer of ∼550 bp was obtained and cloned into pGEM-T Easy. One of the obtained clones was checked by sequencing to confirm the specificity of the primers for XTHs. The deduced amino acid sequence showed an identity of 98% with the previously characterized XTHs of *T. aestivum* (Sequence ID: Sp| Q41542.1| XTH_WHEAT) and *H. vulgare* (Sequence ID: emb| CAA62847), and 97% with *O. sativa* L. (Sequence ID: ref| NP_001068033).

### Effect of Dehydration on XTH Expression, XET Activity, and XET Action

The effects of dehydration on *XTH* expression and XET activity and action were analyzed in the seedlings of the wheat cultivars Simeto, Creso, and Ardente, respectively, sensitive, moderately tolerant and tolerant to dehydration as estimated by the RWC assay.

**FIGURE 3:**
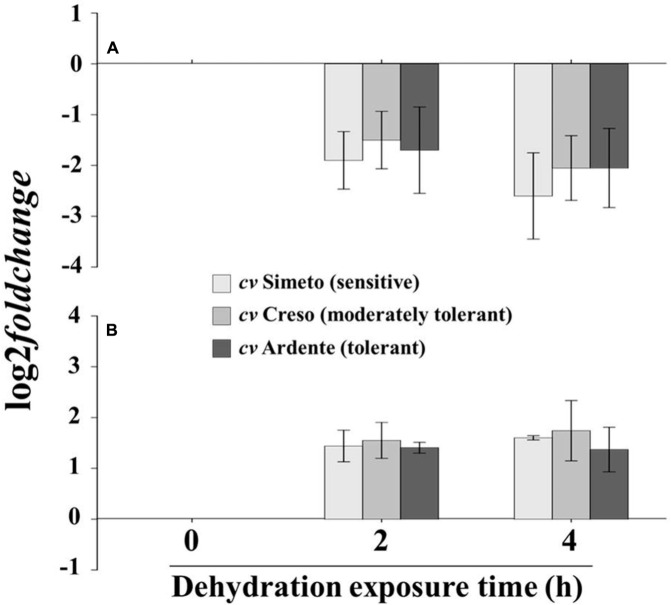
***XTH* expression profile in excised segment extracts of (A)** the first 5 mm of the root apical region and **(B)** the root sub-apical region of 3-day-old wheat seedlings (cultivar Simeto, Creso, and Ardente) subjected to drought stress for 2 and 4 h. XTH expression is reported as decrease in transcript level (log_2_ fold change) with respect to time 0 (control). Data are the mean ± standard deviation of three independent replicates (*n* = 3).

*XTH* expression was quantified by qRT-PCR on the apical (10 mm starting from the root cap) and the sub-apical (next 40 mm) segments of the root (**Figure 1i**) excised from 3-day-old wheat seedlings subjected to dehydration or maintained in well-watered conditions (control) for 0, 2, and 4 h. In the root apical region (**Figure 3A**), 2 h of dehydration caused a small but consistent decrease (log_2_ fold change between -2 and -1; deemed insignificant) in *XTH* expression levels of all analyzed cultivars; after 4 h a slight but significant down-regulation in *XTH* expression was detected in all cultivars, with the highest inhibition found in Simeto (log_2_ fold change = -2.6). In the root sub-apical region (**Figure 3B**) both 2 and 4 h of dehydration caused a small but consistent increase (log_2_ fold change ≈ +1.5; deemed insignificant), in all investigated cultivars.

**FIGURE 4:**
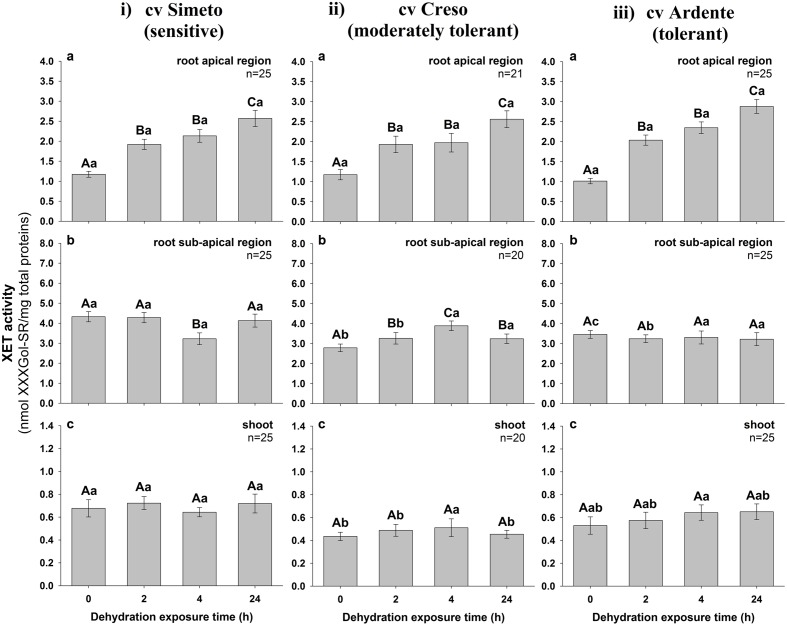
**Extractable XET activity in excised segments of **(a)** root apical region, **(b)** root sub-apical region, and **(c)** shoot from 3-day-old durum wheat (*T. durum* Desf.) seedlings of (i)** Simeto, **(ii)** Creso, and **(iii)** Ardente cultivars, differing in dehydration tolerance. The seedlings of each cultivar were subjected to drought stress for 2, 4, and 24 h. XET activity is expressed as nmol of xyloglucan–XXXGol-SR formed by the transglycosylation reaction, per mg of total protein. The results represent the average of *n* independent replications ± standard error. Different capital letters above bars indicate statistically significant differences (*p* < 0.05) between time points within the same cultivar, while lower case letters indicate statistically significant differences across cultivars in response to stress duration, as determined by the Tukey’s *post hoc* test.

The total extractable XET activity was evaluated by a biochemical assay on crude extracts obtained from the above-mentioned root segments and entire shoots (coleoptile plus leaf) excised from 3-day-old wheat seedlings of the three cultivars subjected or not (control) to dehydration for 2, 4, and 24 h. The values, expressed as nmol of XXXGol-SR transferred to xyloglucans/mg of total protein, are reported in **Figure 4**. Extractable XET activity of the well-watered seedlings was stable over time in all assayed cultivars, with values similar to those measured at zero time point. In the root apical region of the Simeto seedlings subjected to dehydration (**Figure 4ia**) XET activity was significantly (*p* < 0.001, *n* = 25) increased by approx. 64 and 120% after 2 or 4 and 24 h from stress induction, respectively, while a significant (*p* < 0.001, *n* = 25) 30% decrease characterized the sub-apical region of the root exclusively after 4 h of dehydration (**Figure 4ib**). The XET activity of the shoot (**Figure 4ic**) was not significantly affected by dehydration regardless of treatment duration (*p* = 0.555; *n* = 25). With regard to the cultivar Creso, a consistent (63%) and statistically significant (*p* < 0.001, *n* = 21) increase of XET activity was induced in the root apical region after 2 h of dehydration (**Figure 4iia**), which further rose to 110% at the 24 h time point. A significant (*p* < 0.001, *n* = 20) progressive increase in XET activity was also observed in the root sub-apical region with a peak (42%) at 4 h (**Figure 4iib**). In the shoot (**Figure 4iic**), dehydration did not cause statistically significant changes (*p* = 0.336, *n* = 19) of XET activity. In the root apical region of the cultivar Ardente (**Figure 4iiia**), 2, 4, and 24 h of dehydration caused, respectively, a 100, 130, and 180% increase of XET activity compared to the control (p < 0.001, n = 25); in contrast, XET activity was not affected by dehydration in the sub-apical region of the root (*p* = 0.951, *n* = 24; **Figure 4iiib**) or in the shoots (*p* = 0.383, *n* = 16; **Figure 4iiic**) remaining almost constant up to 24 h.

In order to distinguish between extractable XET activity (assayable *in vitro* under ‘optimized’ conditions) and XET action on its endogenous donor substrates *in situ* in the walls of living cells, we performed the *in vivo* estimation of XET action using confocal microscopy techniques according to Vissenberg et al. (2000). This method also gave improved spatial resolution of XET distribution along the root. The analyzed region (**Figure 1ii**) included the root cap (0–0.5 mm), the root meristem (between 0.5 and 1 mm), the zone of cell elongation (between 1 and 2.5 mm), and the zone of cell differentiation (between 2.5 and 5 mm). The fluorescence of sulforhodamine-labeled XXXG stably linked to root endogenous substrates, proportional to XET action, was quantified sequentially in approximately 500-μm portions starting from the root cap and expressed as pixel intensity/0.5 mm^2^.

**FIGURE 5:**
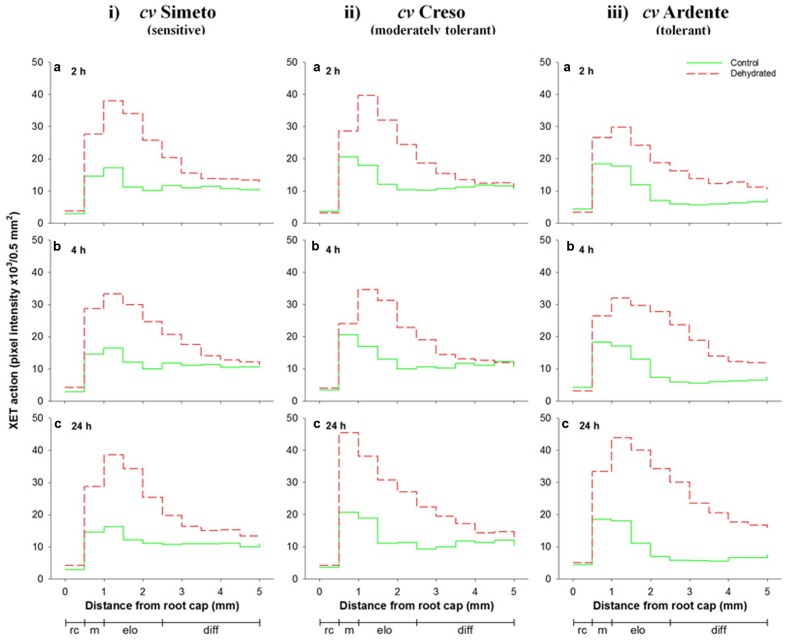
**Sequential *in vivo* XET action on endogenous xyloglucan as donor substrate evaluated by confocal microscopy observations of the first 5 mm root apical region of 3-day-old durum wheat (*T. durum* Desf.) seedlings of (i)** Simeto, **(ii)** Creso, and **(iii)** Ardente cultivars, differing in dehydration tolerance. The seedlings of each cultivar were subjected to dehydration for 2 **(a)**, 4 **(b)**, and 24 h **(c)**. The fluorescence, proportional to the XET action of endogenous XTHs on endogenous xyloglucan, was quantified sequentially in 500-μm segments starting from the root cap, with ImageJ software. The results shown in each graph represent the average of three independent replications (*n* = 3). Standard deviation was below 5%. rc = root cap, m = root meristem, elo = zone of cell elongation, diff = zone of cell differentiation. Zone distribution below the *x*-axis is attributed to control roots; stressed roots may have a different profile of cell development.

**Figure 5** (see also **Supplementary Figure S2**) shows the *in vivo* XET action evaluated sequentially in the root apical region of 3-day-old wheat seedlings (cultivars Simeto, Creso, and Ardente) subjected for 2, 4, and 24 h to dehydration. At root cap level, XET action was unaffected by stress even after 24 h from induction in all wheat cultivars, while differential significant increases were detected in all other regions. In the cultivar Simeto (**Figure 5i**), an 80% increase was detected in the root meristematic zone even after 2 h of dehydration (**Figure 5ia**) and XET action further increased (to 100%) after 24 h (**Figure 5ic**). In the cell elongation zone, 2 h of dehydration caused a 160% increase of XET action, which remained stable for the entire treatment period. In the zone of the root in which the cells are fully elongated and undergoing differentiation, stress caused a slight increase in XET action, with a peak of 40% at 24 h (**Figure 5ic**). In Creso (**Figure 5ii**), at root meristem level, XET action was augmented by 30–40% after 2 and 4 h (**Figures 5iia,b**), reaching 120% after 24 h dehydration with respect to the control, while it was increased by 135 and 60% in the elongation and cell differentiation zones, respectively, after 24 h of dehydration (**Figure 5iic**).

In the cultivar Ardente (**Figure 5iii**), a 50% increase of XET action was observed at root meristem level after 2 and 4 h of stress (**Figures 5iiia,b**), which reached 90% at 24 h (**Figure 5iiic**). In the cell elongation zone, dehydration induced a sharp and sustained increase in action between 110% at 2 h (**Figure 5iiia**) and 240% at 24 h (**Figure 5iiic**). The greatest effect on the XET action was observed in the zone of cell differentiation, where 130, 160, and 300% increases were registered after 2, 4, and 24 h of dehydration, respectively (**Figures 5iiia,b,c**).

### Effects of Heat Treatment on XTH Expression, XET Activity, and XET Action

**FIGURE 6:**
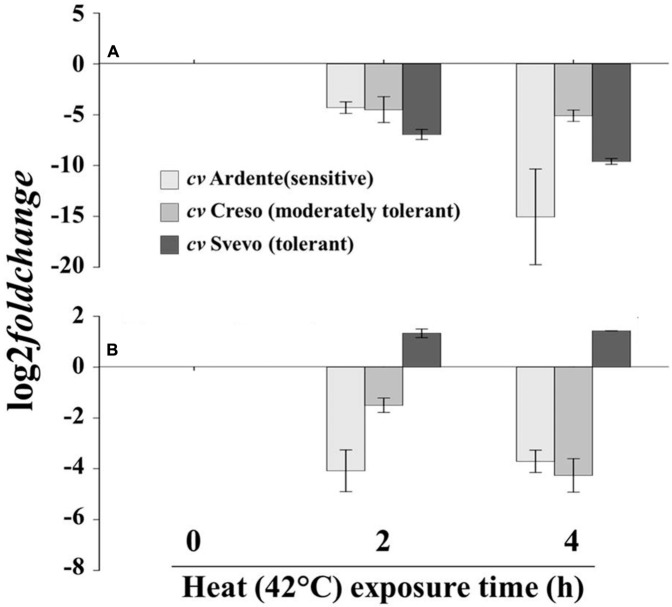
***XTH* expression profile in excised segment extracts of (A)** the first 5 mm of the root apical region and **(B)** the root sub-apical region of 3-day-old wheat seedlings (cultivar Ardente, Creso, and Svevo) subjected to heat stress (42°C) for 2 and 4 h. XTH expression is reported as transcript inhibition level (log_2_ fold change) with respect to time 0 (control). Data are the mean ± standard deviation of three independent replicates (*n* = 3).

The cultivars Ardente, Creso and Svevo, assumed to be, respectively, sensitive, moderately tolerant and tolerant to thermal treatment on the basis of the CMS test (**Figure 2**), were used to assess differences in *XTH* expression and XET activity and action after exposure at 42°C for 0, 2, and 4 h. Assays were performed on the apical (10 mm) and sub-apical (next 40 mm) regions of root segments excised from 3-day-old wheat seedlings as previously described (**Figure 1**). In the root apical region (**Figure 6A**), 2 h at 42°C induced a log_2_-fold change of about -4, -5, and -7 (i.e., down-regulation) of *XTH* gene expression, in the cultivars Ardente, Creso, and Svevo, respectively. After 4 h at 42°C, this down-regulation remained constant in the cultivar Creso, increased slightly in Svevo (to log_2_ = -9) and strongly in Ardente (to log_2_ = -15). In the root sub-apical region of the cultivar Ardente (**Figure 6B**), however, although *XTH* expression was somewhat inhibited after 2 h of thermal treatment (to log_2_ = -4), no biologically significant differences were detected in Creso and Svevo. Interestingly, after 4 h at 42°C, *XTH* expression was inhibited (to log_2_ = -4) in both Ardente and Creso, whereas there was still no significant variation in Svevo.

**FIGURE 7:**
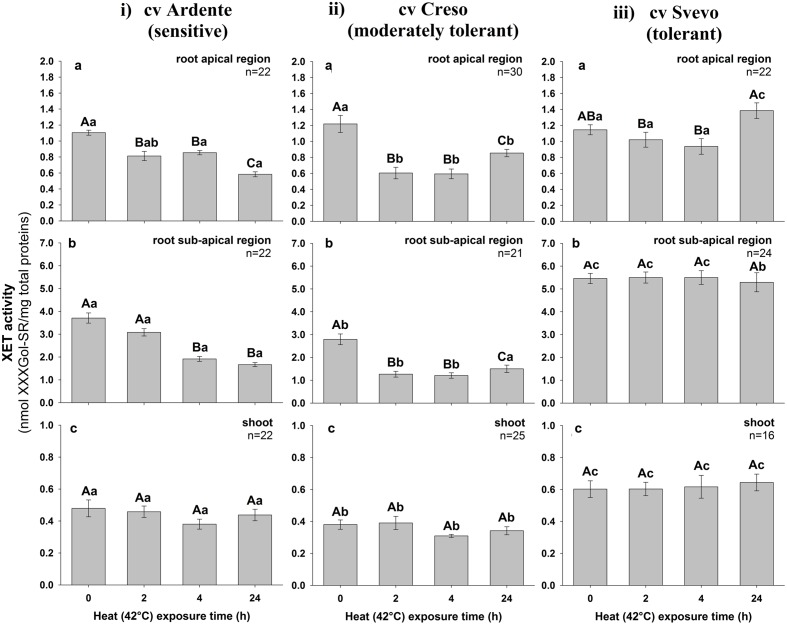
**Extractable XET activity in excised segments of **(a)** root apical region, **(b)** root sub-apical region, and **(c)** shoot from 3-day-old durum wheat (*T. durum* Desf.) seedlings of (i)** Ardente, **(ii)** Creso, and **(iii)** Svevo cultivars, differing in heat tolerance. The seedlings of each cultivar were subjected to heat (42°C) stress for 2, 4, and 24 h. XET activity is expressed as nmol of xyloglucan–XXXGol-SR formed by the transglycosylation reaction, per mg of total protein. The results represent the average of *n* independent replications ± standard error. Different capital letters above bars indicate statistically significant differences (*p* < 0.05) between time points within the same cultivar, while lower case letters indicate statistically significant differences across cultivars in response to stress duration, as determined by the Tukey’s *post hoc* test.

The total extractable XET activity was assayed in crude extracts from the apical and sub-apical root segments and shoots excised from 3-day-old wheat seedlings incubated for 0, 2, 4, and 24 h at 22°C (control) or 42°C (heat stressed). In control conditions, no differences were detected over time in extractable XET activity regardless of cultivar and topological region; in contrast, it appeared differentially influenced by the thermal treatment. In the cultivar Ardente (**Figure 7i**), a progressive highly statistically significant (*p* < 0.001; *n* = 22) decrease in extractable enzyme activity was evidenced at both root apical (-26% after 2 h and -48% after 24 h at 42°C) and sub-apical level (-16, -49, and -54%, after 2, 4, and 24 h at 42°C, respectively; **Figures 7ia,b**) but not in the shoot (*p* = 0.150, *n* = 22; **Figure 7ic**). A similar trend characterized the cultivar Creso (**Figure 7ii**), where heat exposure significantly decreased the extractable XET activity all along the root (*p* < 0.001), but not in the shoot (*p* = 0.148, *n* = 25). However, in this case, inhibition was not progressive but XET activity recovered over time in both apical (-50% after 2 and 4 h and -30% after 24 h at 42°C) and sub-apical (-55% after 2 and 4 h and -45% after 24 h at 42°C) regions (**Figures 7iia,b**). In Svevo (**Figure 7iii**), heat exposure caused statistically significant changes in XET activity only in the root apical region (*p* < 0.001, *n* = 22) where an increase of 19% was registered after 24 h (**Figure 7iiia**), while in the sub-apical region of the root (**Figure 7iiib**) and in the shoot (**Figure 7iiic**) enzyme activity remained almost unchanged even after 24 h of thermal treatment (*p* = 0.941, *n* = 24; *p* = 0.884, *n* = 16, respectively).

**FIGURE 8:**
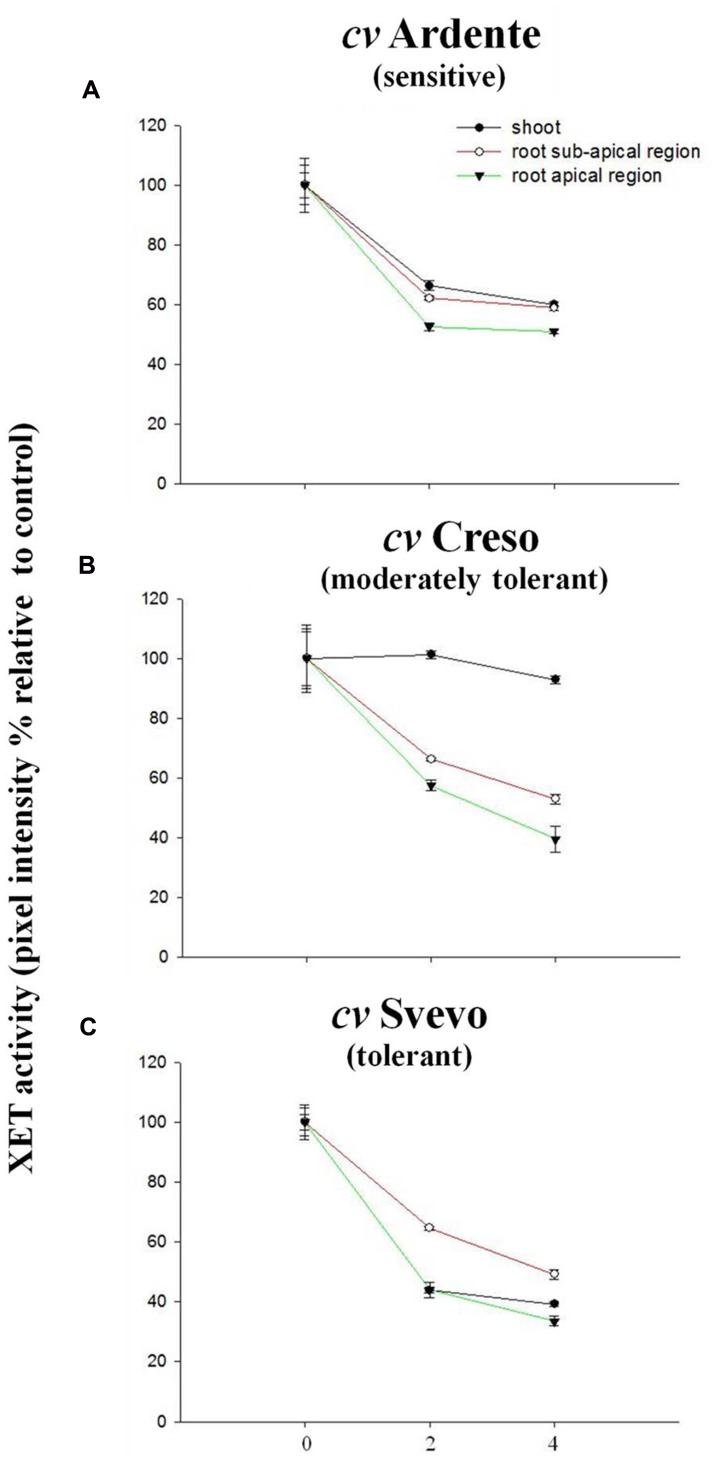
**Effect of a 42°C pre-treatment, applied to enzyme extracts *in vitro*, on their residual XET activity (subsequently assayed at 25°C).** The extracts had been prepared from (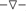) root apical regions, (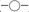) root sub-apical regions, and (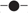) shoots of non-stressed 3-day-old wheat seedlings. Three cultivars were compared: **(A)** Ardente, **(B)** Creso, and **(C)** Svevo, which differ in heat tolerance. XET activity, remaining in the extract after the *in vitro* heat pre-treatment, is given as the fluorescence intensity of the generated xyloglucan–XXXGol-SR expressed as a percentage of that produced by the corresponding untreated extracts. Data are the mean ± standard deviation of six independent replicates (*n* = 6).

The activity of XTH isoforms is differentially influenced by temperature (Purugganan et al., 1997; Steele and Fry, 2000). Therefore, the heat-lability of the extracted enzymes was evaluated. Crude extracts obtained from the apical and sub-apical root regions and shoots excised from 3-day-old non-heat-stressed wheat seedlings were heated at 42°C for 2 or 4 h and then assayed for XET activity. Variations were expressed as percentage with respect to fresh (time 0) extracts which were arbitrarily assigned an XET activity value of 100%. In general, a differential heat-sensitivity of XTH enzymes from different wheat seedlings regions was revealed. In the extracts obtained from the shoots and both root apical and sub-apical regions of the cultivar Ardente (**Figure 8A**) *in vitro* treatment at 42°C for 2 h caused an inhibition of the enzymatic activity of approximately 35, 38, and 48%, respectively. These values were constant even after 4 h at 42°C. In the extracts from the shoot of the cultivar Creso only a negligible loss of XET activity was observed, while there was a 55 and 40% inhibition, respectively, in the root apical and sub-apical regions after 4 h at 42°C (**Figure 8B**). A greater heat-lability (between 50 and 65% loss of activity) was evident in all extracts obtained from the thermo-tolerant cultivar Svevo (**Figure 8C**).

**FIGURE 9:**
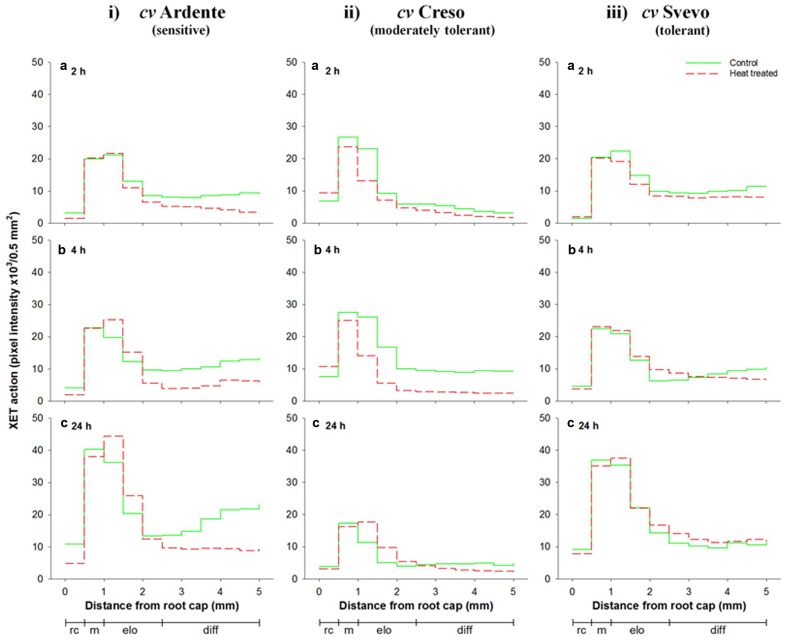
**Sequential *in vivo* XET action on endogenous xyloglucan as donor substrate, evaluated by confocal microscopy observations of the first 5 mm root apical region of 3-day-old durum wheat (*T. durum* Desf.) seedlings of (i)** Ardente, **(ii)** Creso, and **(iii)** Svevo cultivars, differing in heat tolerance. The seedlings of each cultivar were subjected to heat (42°C) stress for 2 **(a)**, 4 **(b)**, and 24 h **(c)**. The fluorescence, proportional to the XET action of endogenous XTHs on endogenous xyloglucan, was quantified sequentially in 500-μm segments starting from the root cap, with ImageJ software. The results shown in each graph represent the average of three independent replications (*n* = 3). Standard deviation was below 5%. rc = root cap, m = root meristem, elo = zone of cell elongation, diff = zone of cell differentiation. Zone distribution below the *x*-axis is attributed to control roots; stressed roots may have a different profile of cell development.

**Figure 9** (see also **Supplementary Figure S3**) shows the *in vivo* XET action evaluated sequentially in the first 5 mm of the root apical region of 3-day-old wheat seedlings of the three cultivars incubated subjected at 42°C for 2, 4, and 24 h. In the root cap of the cultivar Ardente, XET action was progressively inhibited by up to ∼60% after 24 h at high temperature (**Figure 9ic**). At the level of root meristematic zone, XET action was almost identical in control and stressed roots even after 24 h (**Figures 9ia,b,c**), while in the cell elongation zone it was inhibited by 25% after 2 h (**Figure 9ia**), but promptly recovered after 4 h of stress (**Figure 9ib**), to increase (by 25% relative to time 0) after 24 h (**Figure 9ic**). In the root cell differentiation zone a significant (*p* < 0.05) and progressive decrease in the XET action was observed in stressed seedlings. This decrease was approximately 50% after 24 h of thermal treatment compared to control (**Figure 9ic**). The *in vivo* XET action of the cultivar Creso is shown in **Figure 9ii**. In the root cap, XET action was slightly higher in 2 and 4 h stressed seedlings than control (**Figures 9iia,b**), while differences between stressed and unstressed root seedlings were not statistically significant at 24 h (**Figure 9iic**). At root meristem level, thermal treatment induced only a slight variation in the XET action even after 24 h (**Figures 9iia,b,c**). In the region of cell elongation XET action was inhibited by 40 and 50% (**Figures 9iia,b**) after 2 and 4 h at 42°C, respectively, while a 60% increase was registered after 24 h compared to control (**Figure 9iic**). In the cell differentiation zone heat exposure induced an inhibition of XET action, with a maximum of 60% after 4 h which diminished to 20% after 24 h (**Figures 9iia,b,c**). In the cultivar Svevo (**Figure 9iii**), the *in vivo* XET action was unaffected by heat in the root cap, meristematic, and differentiation zones throughout the incubation period (24 h; **Figures 9iiia,b,c**), but in the elongation zone of the root a 20% decrease was observed after 2 h (**Figure 9iiia**) and a negligible (∼10%) increase was registered after 4 and 24 h at 42°C (**Figures 9iiib,c**).

## Discussion

Despite xyloglucans often being minor wall components in the Poaceae (Carpita, 1996; Popper and Fry, 2004), XET activity tends to be extremely high (Fry et al., 1992; Wu et al., 1994). The endogenous transglycanases of *H. vulgare* are incapable of using mixed-linkage β-glucans instead of xyloglucans as donor substrate (Mohler et al., 2013; unlike the case with the fern-ally, *Equisetum*). An explanation for this apparent paradox may be provided by the “biomechanical hotspot” hypothesis recently proposed by Park and Cosgrove (2015). In their model the control of wall extensibility occurs by cleavage of a limited fraction (<1%) of the total xyloglucan that is tightly interlaced with cellulose in specific regions (biomechanical hotspots) where multiple microfibrils come into close contact; thus the high XET activities registered in commelinoid monocots may have a role, along with expansins and hydrolases, in finely regulating cell wall expansion by loosening the constraints on the movement of cellulose microfibrils at these hotspots. Nonetheless, it is noteworthy that in a previous study we have reported a surprisingly high amount of xyloglucans (23–39 mol% of the total cell wall polysaccharides) in the apical root zone of Capeiti and Creso durum wheat seedlings grown under water-stressed and unstressed conditions (Leucci et al., 2008), suggesting an important role of xyloglucans as load-bearing components even in the type II primary cell wall of monocots, at least during cell expansion and differentiation. Likewise, in the walls of rapidly growing maize cell-suspension culture, ∼37% of the total xylose residues are in xyloglucans rather than β-xylans (Kerr and Fry, 2003).

The XTH expression levels detected in the root apical region of wheat seedlings subjected to dehydration gave evidence for a slight but significant down-regulation in all investigated cultivars, with the highest inhibition (log_2_ effect ≈-2.6 with respect to control) found in the sensitive cultivar Simeto. In the root sub-apical region, however, dehydration did not induce any significant variation of the *XTH* expression, irrespective of treatment duration. A consistent down-regulation in the expression of different XTH isoform genes (*XTH6. XTH9. XTH15*, and *XTH16*) has been previously reported in early developing leaf tissue of six natural variants of *Arabidopsis* subjected to mild or severe drought stress by Clauw et al. (2015), whereas an up-regulation of XTH coding genes has been reported in the elongation zone of drought-stressed rice roots (Yang et al., 2006), in water-stressed leaves of hot pepper plants (*C. annuum* L.; Cho et al., 2006) and in osmotically stressed leaves of *Leymus mollis* (Trin.) Pilg., a wild relative of wheat (Habora et al., 2012). Further, the expression behavior of *OsXET9* was differently affected by drought in the Pei’ai64s rice cultivar depending on tissue and development stage, being up-regulated exclusively in the panicles of heading and flowering stage plants (Dong et al., 2011). Different expression patterns of *XTH* genes were also found in shoots and roots of *Arabidopsis* plants subjected to 24 h of drought stress by microarray determination, with differential rates among the family members (Tenhaken, 2014). Thus, a highly specific spatial regulation of *XTH* genes could contribute to strengthening or loosening the cell wall in well-defined topological regions of the plant contributing to susceptibility/tolerance to dehydration. In support of this, using a proteomic approach, Zhu et al. (2007) revealed major changes in the abundance of a large number of cell wall proteins, including XTHs, based on their spatial distribution within the primary root elongation zone of maize seedlings subjected to water deficit. Specifically, of the four putative XTHs identified, two were quantitatively reduced in the first 3 mm of the root apical region, one decreased along the whole root and the last exhibited a slight increase in abundance mainly in the 3–7 mm root sub-apical region after drought treatment.

In spite of the significant down-regulation of *XTH* genes, the extractable XET activity assayed *in vitro* increased considerably with stress exposure time in the root apical region of all assayed cultivars. The greatest increase was recorded in the tolerant cultivar Ardente (180%), while in Simeto and Creso it was 110%. These increases in extractable activity were mirrored by increases in XET action, monitored *in vivo*. Major differences were observed in the root cell elongation and differentiation zones, in compliance with the cultivars’ dehydration tolerance degree. The increase of XET activity and action recorded in the root apical region may cause (or result from) an increased division rate of the meristematic cells and/or a controlled loosening of cell wall polymers in the zone of expansion growth. Both these events could support root growth and development even at low water potential, representing an important mechanism of drought tolerance which allows the stressed plants to invest available resources into soil exploration for residual water (Spollen et al., 1993; Tenhaken, 2014). Similar results were reported by Wu et al. (1994) in the apical few millimeters of maize primary roots growing at low (-1.6 MPa) water potential. The authors reported that the enhancement of the total extractable XET activity was greater immediately behind the apex (0–5 mm) than in following (5–10 mm) sub-apical region of water-stressed compared to well-watered roots, suggesting that XTH may be involved in the stress-induced enhancement of cell wall extensibility in the apical region. This is supported by the work of Sharp et al. (2004) reporting the persistence of root growth rate in the first 3 mm of maize roots subjected to drought stress, while the cells in next 3–4 mm were characterized by a progressive decrease of elongation rate. A differential inhibition of coleoptile (∼75%) and root (∼50%) growth has also been reported in durum wheat seedlings (cultivars Creso and Capeiti) incubated in the presence of a 20% solution of PEG 4000 (Ψ_w_ - 0.5 MPa) for 24–48 h (Piro et al., 2003). Another possible explanation for the maintenance of XET action in spite of the down-regulation of XTH genes may also be related to an increased supply of donor substrates for the smaller number of XTH enzyme molecules still able to perform endotransglucosylation reactions. This may be achieved by up-regulation of the enzymes involved in the synthesis of these precursors and/or by their increased secretion in stress conditions.

In the root sub-apical region a slight increase in the XET activity in response to dehydration was observed only in the cultivar Creso, while in the cultivar Simeto the 30% transient decline measured after 4 h from stress induction recovered to control values at 24 h. In the cultivar Ardente, stress did not seem to induce any change in XET activity. The maintenance of XET activity at the level of root sub-apical region subjected to dehydration may not be correlated to the rate of root growth, which is greatly reduced in this zone (Sharp et al., 2004), but to the initiation and tip growth of root hairs where XTHs are greatly involved (Vissenberg et al., 2001). Root hairs play an important role in water and mineral absorption and create resistance between the root and the soil particles, facilitating deep penetration by the root apex toward better hydrated areas (Peterson and Farquhar, 1996).

With respect to thermal treatment, in the root apical region, XTH expression was down-regulated in all wheat cultivars and particularly in the thermo-sensitive cultivar Ardente. A strong down-regulation was also noted in the root sub-apical region of the sensitive and moderately tolerant cultivars Ardente and Creso but not in the tolerant cultivar Svevo. In a different approach, a transcriptomic study performed on Chinese cabbage (*Brassica rapa*, L.) showed that several genes, encoding proteins from the XTH family, were up-regulated following heat treatment (Yang et al., 2006), indicating that plant response to stress is highly complex and often depends on species, genotype, plant age, and organ, as well as on timing and intensity of stress application. The results of *XTH* expression in Ardente and Creso are consistent with the decreased XET *in vitro* activity values measured at the level of root apical region, while they partially clash in the cultivar Svevo. A large number of XTH isoforms with different distribution and physiological roles are known to be differentially affected by stress (Campbell and Braam, 1999b; Sharmin et al., 2012). This means that, in stress conditions, specific XTH isoforms are up-regulated while other are down-regulated according to the proposed redundant function of *XTH* genes, whereby the repression of one member may be compensated for by the up-transcription of the others (Matsui et al., 2005).

In the cultivar Ardente (thermo-sensitive), the *in vitro* XET activity was significantly inhibited at both root apical (>45%) and sub-apical (>50%) level after 24 h at 42°C. Such inhibition is reduced to 30 and 45%, respectively, in the root apical and sub-apical regions in the cultivar Creso (moderately tolerant) and is not significant, or even increased (19%), in the thermo-tolerant cultivar Svevo. No difference in XET activity was observed in the shoots of all investigated cultivars. It is well established, in fact, that roots are more sensitive to heat than the aerial part of the plant, with high soil temperature more detrimental than high air temperature for whole plant growth (Xu and Huang, 2000; Le Gall et al., 2015).

The *in vivo* experiments showed a general reduction of XET action in the walls of living cells of thermal treated root apical segments, mirroring the observed change in extractable XET activity assayed *in vitro*, and highlighted interesting differences at microscale level. While in the cultivar Svevo XET action was not affected by thermal treatment all along the root apical region, in Ardente and Creso it was almost stable in the meristematic zone, increased in the elongation zone (25 and 60%, respectively) and decreased (50 and 20%, respectively) in the zone of cell differentiation after 24 h at 42°C, indicating an intraspecific variability in the sensitivity of XET action to high temperatures, also depending on the particular topological region of the root. Further, the localized increase/decrease of XET action in the root apical region of the heat sensitive and moderately tolerant cultivars could represent an acclimation response to high temperature realized through the redirection of root growth to diminish stress exposure (Potters et al., 2007). Thus, the increase observed within the cell expansion zone may support root growth to reach deep soil, while the decrease within the zone of cell differentiation may reduce the root surface exposed to high temperature which could potentially interfere with root hair development. This agrees with the observation that living epidermal cells of *Arabidopsis* subjected to short-term heat shock show a temporary disruption of cytoskeletal elements that are important for plant cell growth and morphogenesis including trichome development and tip growth of root hairs (Müller et al., 2007). The stable, or even slight increase, of XET activity and action observed in thermo-tolerant cultivar Svevo could instead support the growth of the root system ensuring the correct development of whole plant even at high temperature, constituting a fundamental mechanism in the process of heat-tolerance (Huang et al., 2012). An increased level of *XTH* transcripts has been reported in ripening grapevine (*Vitis vinifera* L.) fruits following heat stress and related to the need for more flexible cell walls to allow an adaptation of berry volume to temperature (Rienth et al., 2014). It has also been speculated that, after cell elongation has ceased, the functional significance of XTHs could be linked to the strengthening of cell walls by increasing xyloglucan polymerization, which might help plants to adapt to high temperature by reinforcing of connections between primary and secondary walls (Yang et al., 2006).

Studies carried out on two species of forage plants of the genus *Agrostis* with different heat stress susceptibility showed that, when exposed to high temperature, the tolerant species (*A. scabra* Willd.) maintained better root growth than the thermo-sensitive (*A. stolonifera* L.; Lyons et al., 2007). The differential change of XET activity and action observed in the Svevo, Creso, and Ardente seedlings incubated 42°C could, therefore, be related to mechanisms similar to those described in *Agrostis*.

Several XTH isoforms with different temperature optima in a range between 18 and 37°C have been identified in *Arabidopsis* (Campbell and Braam, 1999a), and an isoform exhibiting continued activity at 4°C was also reported (Purugganan et al., 1997). Similar results were obtained by Steele and Fry (2000), who measured the XET activity of various XTH isoforms isolated from seedlings of bean (*Vigna radiata* L. Wilczek) and shoots of cauliflower (*Brassica oleracea* L.) and subsequently exposed *in vitro* to 42°C, showing an inhibition between 55 and 80% compared to their maximal efficiency. Thus, the XTHs extracted from the various topological regions of the seedling of the three durum wheat cultivars have an optimal temperature stability different from 42°C, at which a decrease of over 40% in XET activity was measured. An exception was the extract obtained from the shoot of the cultivar Creso, where XET activity was not affected by heat. The higher heat-sensitivity of the enzyme in the extracts of the thermo-tolerant cultivar Svevo than in those of the moderately tolerant and sensitive cultivars Creso and Ardente, in contrast with the *in vitro* determinations, are probably due to response mechanisms adopted by living cells to improve the thermal stability of XTHs. Soto et al. (1999) reported a direct correlation between the heterologous expression of a plant small heat shock protein (HSP17.5 from *Castanea sativa* Miller) and the thermo-stability of soluble proteins in heat-stressed transgenic *Escherichia coli*. Heat shock proteins are mainly intraprotoplasmic, but some were also detected in the cell wall of barley coleoptiles and roots subjected to heat stress, and this has suggested a role in wall protein protection (Leone et al., 2000). An HSP90 was localized by confocal microscopy in the cell wall of *Aspergillus fumigatus* subjected to heat stress conditions, indicating that these proteins, in non-physiological conditions, can be released into the apoplast (Lamoth et al., 2012).

## Conclusion

Our results indicate that dehydration and heat stress, applied singularly, differentially influence the *XTH* expression profiles and the activity and action of XET in wheat seedlings, depending on the degree of susceptibility/tolerance of the cultivars. In all investigated cultivars the root apical region was the district mainly affected by both stresses. Significantly, at shoot level, neither dehydration nor heat treatment induced a change in XET activity or action irrespective of the degree of susceptibility/tolerance. This indicates that the variations in XTH expression level and XET activity and action are implemented mainly at root apical region, confirming the pivotal role of this organ in stress perception and signaling to the whole organism. In addition, the data related to the cultivars Ardente and Creso, common to both dehydration and heat treatments, showed conflicting effects depending on stress type: dehydration determined an overall increase, at least in the apical region of the root, of XET activity and action, while heat generally induced a decrease. This suggests a different involvement of XTH enzymes in the mechanisms of abiotic stress responses. Consequently, further research on the effects of the simultaneous exposure to dehydration and heat is required to expand our knowledge of the role of XTHs in adaptation and acclimation of crop plants to stress conditions naturally occurring in open fields.

## Author Contributions

AI: Performed most of the biochemical experiments and contributed to the first draft of the manuscript. MDC: performed the microscopical observations. ES and LD: Contributed to the expression experiments and critically discussed the obtained results. MDP, PR, and CP performed the experiments and critically discussed the results on wheat susceptibility/tolerance to drought and high temperatures. ML: Conceived and designed the experiments and wrote the manuscript with the fundamental help of GD, GP, and SF. All authors contributed to the discussion and approved the final manuscript.

## Conflict of Interest Statement

The authors declare that the research was conducted in the absence of any commercial or financial relationships that could be construed as a potential conflict of interest.

## Footnotes

1 http://au.expasy.org/tools/dna.html

2 http://www.lifetechnologies.com/

3 http://www.megazyme.com

## Supplementary Material

**FIGURE S1 | Melting-curves of the XTH quantitative real-time PCR products after 35 cycles obtained from root segments excised from 3-day-old wheat seedlings subjected to** **(A)** control conditions, **(B)** drought, or **(C)** heat (42°C) stress. The curves obtained from the root apical and sub-apical regions subjected to stress for 2 and 4 h of a given cultivar are superimposed in each graph.

**FIGURE S2 | Confocal microscopy observations of the *in vivo* XET action of endogenous XTHs on endogenous xyloglucans in the first 5 mm root apical region of 3-day-old seedlings of three durum wheat cultivars:** **(i)** Simeto, **(ii)** Creso, and **(iii)** Ardente, differing in dehydration tolerance. The seedlings of each cultivar were subjected to dehydration for 2, 4, and 24 h. The images are representative examples of three independent replications. rc = root cap, m = root meristem, elo = zone of cell elongation, diff = zone of cell differentiation.

**FIGURE S3 | Confocal microscopy observations of the *in vivo* XET action of endogenous XTHs on endogenous xyloglucans in the first 5 mm root apical region of 3-day-old seedlings of three durum wheat cultivars:** **(i)** Ardente, **(ii)** Creso, and **(iii)** Svevo, differing in heat tolerance. The seedlings of each cultivar were subjected to heat (42°C) stress for 2, 4, and 24 h. The images are representative examples of three independent replications. rc = root cap, m = root meristem, elo = zone of cell elongation, diff = zone of cell differentiation.
