# Supplementary figures and images for: Drought and Heat Differentially Affect XTH Expression and XET Activity and Action in 3-Day-Old Seedlings of Durum Wheat Cultivars with Different Stress Susceptibility
Source: Front Plant Sci. 2016 Nov 10;7:1686. doi: 10.3389/fpls.2016.01686 (PMC5102909; doi:10.3389/fpls.2016.01686)

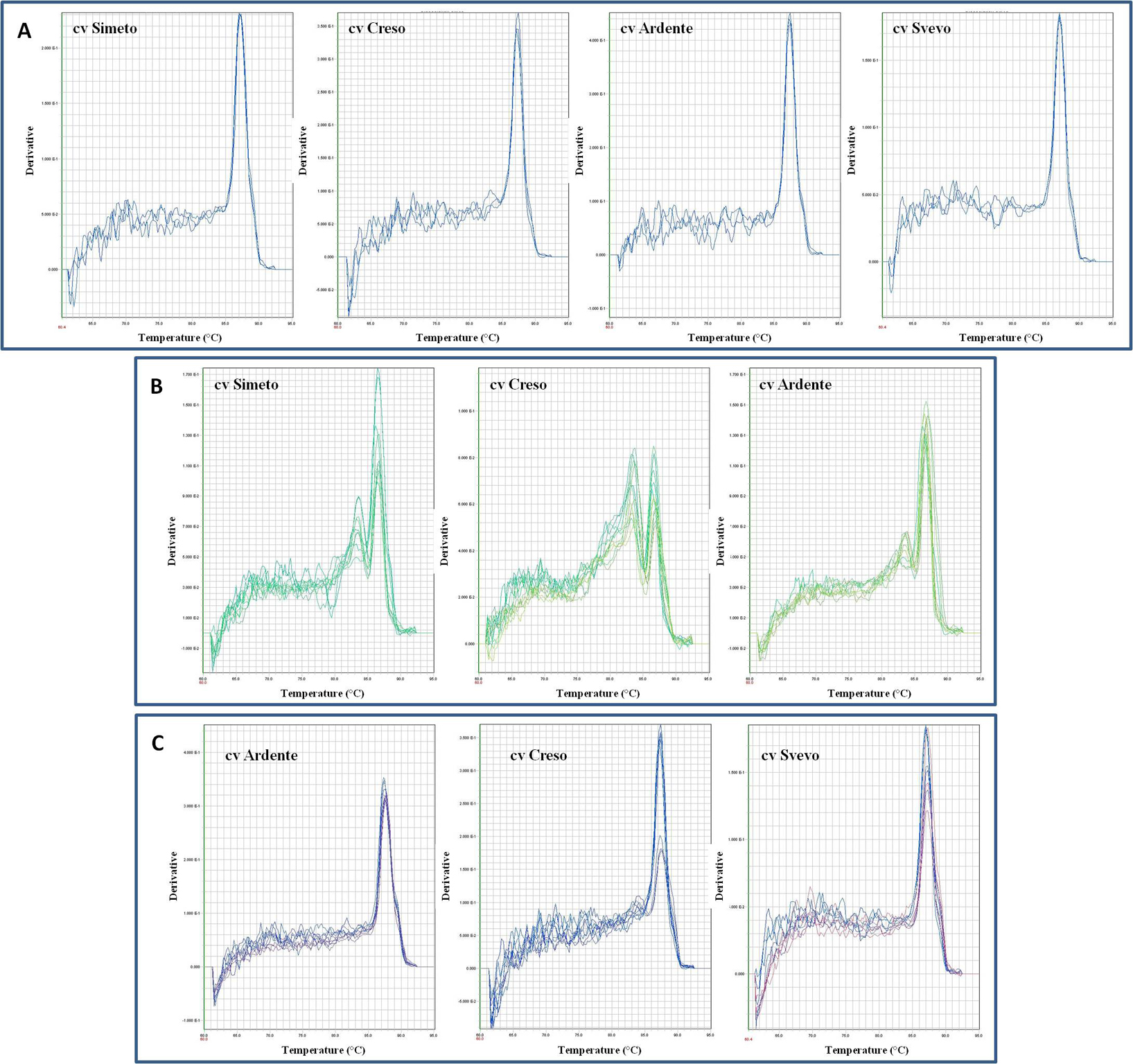

Supplement: Supplementary file 1 [file Image_1.JPEG]

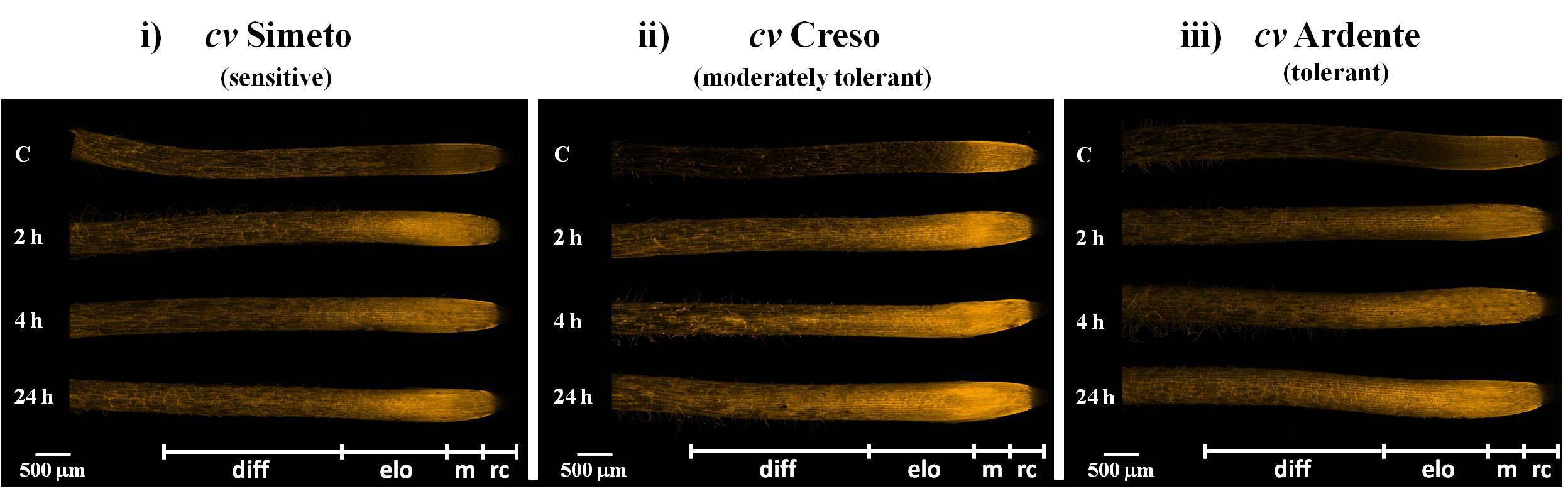

Supplement: Supplementary file 2 [file Image_2.JPEG]

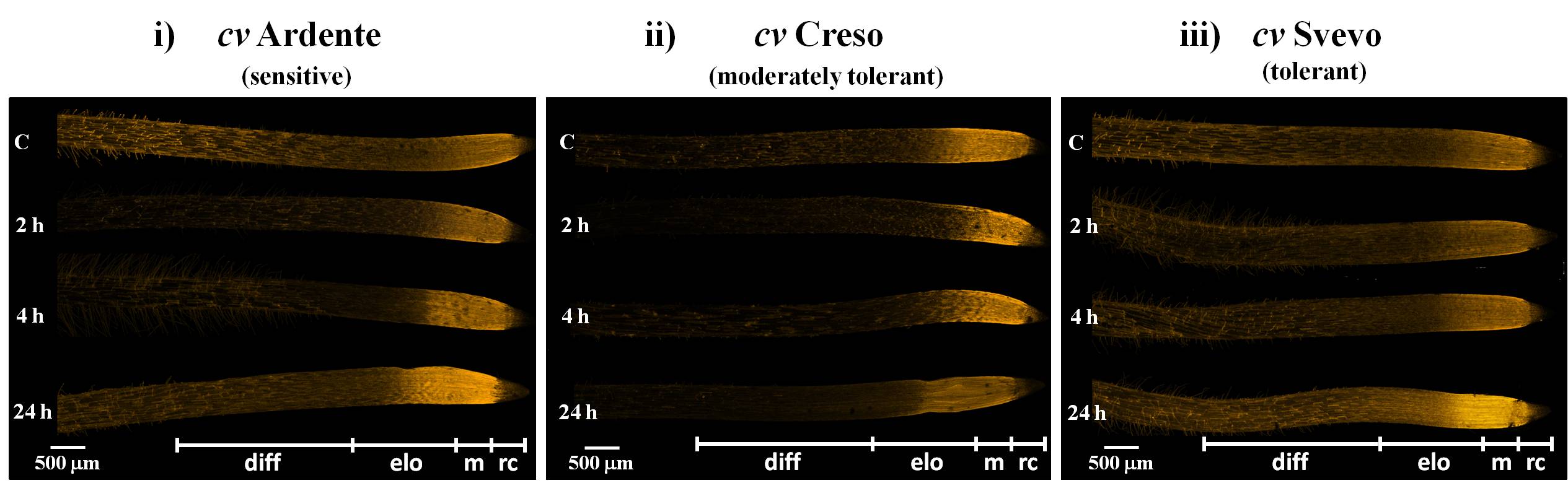

Supplement: Supplementary file 3 [file Image_3.JPEG]
