# Supplementary material for: Drought and Heat Differentially Affect XTH Expression and XET Activity and Action in 3-Day-Old Seedlings of Durum Wheat Cultivars with Different Stress Susceptibility
Source: Front Plant Sci. 2016 Nov 10;7:1686. doi: 10.3389/fpls.2016.01686 (PMC5102909; doi:10.3389/fpls.2016.01686)
Supplement: Supplementary file 4 [file Table_1.DOCX]

**Supplementary table 1:** Oligonucleotides used for RT-PCR.

| **Primer** | **Sequences (5’-3’)** |
| --- | --- |
| XTH-for | 5’-cggcgggcgggaggtgcagc-3’ |
| XTH-rev | 5’-ggaagccccggtaggaggc-3’ |
| Tub-for | 5’-accgccagctcttccaccct-3’ |
| Tub-rev | 5’-tcactggggcataggaggaa-3’ |
